# Comprehensive transcriptomic analyses of tissue, serum, and serum exosomes from hepatocellular carcinoma patients

**DOI:** 10.1186/s12885-019-6249-1

**Published:** 2019-10-28

**Authors:** Robin Mjelle, Simona O. Dima, Nicolae Bacalbasa, Konika Chawla, Andrei Sorop, Dana Cucu, Vlad Herlea, Pål Sætrom, Irinel Popescu

**Affiliations:** 1 Department of Clinical and Molecular Medicine, Norwegian University of Science and Technology, NTNU, Erling Skjalgssons gt 1, 7030 Trondheim, Norway; 2 Department of Computer Science, Norwegian University of Science and Technology, NTNU, Trondheim, Norway; 3 Center of Digestive Diseases and Liver Transplantation, Fundeni Clinical Institute, Bucharest, Romania; 4 Center of Excellence in Translational Medicine, Fundeni Clinical Institute, Bucharest, Romania; 5 Bioinformatics Core Facility-BioCore, Norwegian University of Science and Technology, NTNU, Trondheim, Norway; 6 Department of Anatomy, Physiology, and Biophysics, Faculty of Biology, University of Bucharest, Bucharest, Romania; 7 K.G. Jebsen Center for Genetic Epidemiology, Norwegian University of Science and Technology, NTNU, Trondheim, Norway; 8 Acad. Nicolae Cajal Institute of Medical Scientific Research, Titu Maiorescu University, Bucharest, Romania

**Keywords:** HCC, microRNA, Gene expression, Exosomes, Serum

## Abstract

**Background:**

The expression of microRNAs (miRNAs) is a promising prognostic and diagnostic tool in hepatocellular carcinoma (HCC). Here we performed small RNA sequencing (sRNA-seq) of tissue, serum and serum exosomes to investigate changes in miRNA expression between the different sample types and correlated the expression with clinical parameters. We also performed gene expression arrays on tumor and normal tissue.

**Results:**

Paired tissue, serum and serum exosomes sequencing revealed consistent positive correlation of miR-21 between serum exosomes and tumor tissue, indicating that miR-21 could be exported from tissue to circulation via exosomes. We found that let-7 miRNAs are generally upregulated in serum exosomes compared to whole serum, indicating that these miRNAs could be preferentially loaded into exosomes. Comparing serum from HCC patients with serum from healthy individuals revealed a global increase of miRNAs in serum from HCC patients, including an almost 4-fold increase of several miRNAs, including the liver-specific miR-122. When correlating miRNA expression with clinical parameters we detected significant association between hepatitis B virus (HBV) infection and miR-122 in serum as well as several serum and tissue-miRNAs that correlated with surgery type. We found that miR-141 and miR-146 correlated with cirrhosis in tumor tissue and normal tissue, respectively. Finally, high expression of miR-21 in tumors were associated with poor survival. Focusing on gene expression we found several significant messenger RNAs (mRNAs) between tumor and normal tissue and a Gene Ontology (GO) analysis revealed that these changes were mainly related to cell cycle and metabolism. Further, we detected mRNAs that correlated with cirrhosis and HBV infection in tissue. Finally, GO analysis of predicted targets for miRNAs down-regulated in tumor found that these were enriched for functions related to collagen synthesis.

**Conclusions:**

Our combined data point to altered miRNA and mRNA expression contributing to both generally impaired lipid metabolism and increased cell proliferation and a miRNA-driven increase in collagen synthesis in HCC. Our results further indicate a correlation in miRNA expression between exosomes, serum, and tissue samples suggesting export from tumors via exosomes. This correlation could provide a basis for a more tumor-specific miRNA profile in serum.

## Background

Hepatocellular carcinoma (HCC) is one of the most common cancers worldwide with more than 780,000 new cases in 2012 (http://globocan.iarc.fr/Pages/fact_sheets_cancer.aspx). Common risk factors of HCC include alcohol consumption, hepatitis B virus (HBV), hepatitis C virus (HCV), and non-alcoholic fatty liver disease. Circulating biomarkers in serum and exosomes, such as microRNAs (miRNAs) and other small non-coding RNAs (ncRNAs) are promising tools for discovering and monitoring HCC [1–5]. For example, levels of miR-21 in serum exosomes are significantly higher in patients with HCC compared to controls [6, 7]. In whole serum, several studies have identified potential diagnostic miRNAs [8–14]; however, no consensus has been reached on a panel of miRNAs that robustly identifies HCC at an early stage of the disease. In tissue, several miRNAs are found to be dysregulated between tumor and normal tissue [15–19], for instance miR-21, miR-199, and miR-221. Different miRNAs are shown to correlate with the main risk factors of HCC, including liver cirrhosis [20], HCV [21, 22] and non-alcoholic fatty liver disease [23], indicating that miRNAs have a diagnostic potential in detecting HCC in an early phase of the disease. Other small non-coding RNAs, including small nucleolar RNAs (snoRNAs) and transfer RNAs (tRNAs) are abundant in HCC and have been implicated in the tumorigenesis of HCC [24–26].

MicroRNAs can be actively sorted into exosomes in complex with Argonaute (Ago) proteins. These exosomes can be released into circulation from apoptosis or from active export mechanisms [27] and may provide more tumor specific miRNA profiles than whole blood and serum. Specific exosomal miRNA profiles have been identified in HCC [2, 4, 28]. Furthermore, exosomes can transport miRNAs between cells, as shown for miR-122 between Huh7 and HepG2 human liver cancer cell lines [29] and for miRNAs between human and mouse liver cells and primary B cells [30]. This exosome-mediated transport can lead to the transported miRNAs targeting messenger RNAs (mRNAs) in the recipient cells. For example, exosomes containing miR-122 can lead to increased sensitivity to chemotherapy due to down-regulation of miR-122 target genes [31].

Here we investigated differences in miRNA expression in tissue, serum, and exosomes of HCC patients by sequencing small RNAs (sRNA-seq) in tumor tissue, normal tissue, serum exosomes, and whole serum from the same patient. We found that miRNAs upregulated in tumor tissue, including miR-21, were upregulated in serum exosomes, indicating that exosomes of tumor origin are enriched among serum exosomes. Further, we correlated miRNA and mRNA expression from HCC tissue and serum with clinical parameters and detected miRNAs and mRNAs associated with Cirrhosis and HBV.

## Methods

### Sample material

A total of 89 patients with primary HCC who underwent a curative liver resection and 56 patients who underwent liver transplantation at Fundeni Clinical Institute, Bucharest, Romania were included in this study. Liver tumor samples and adjacent non-tumor tissue were collected at the time of surgery in a stabilizing solution RNAlater (Sigma, St. Louis, MO), and stored at − 80 °C until collection of all samples. Plasma and serum aliquots were prepared from blood samples obtained prior to surgery and were stored at − 80 °C.

The study conformed to the ethical guidelines of the 1975 Declaration of Helsinki and was approved by the Ethics Committee of the Fundeni Clinical Institute (30,884/22.10.2014). All patients signed a written informed consent. Follow-up was completed on 25 August 2016. The period of follow up was defined from the date of surgery to the date of patient’s death or the last follow-up point.

### RNA and exosome isolation

For the small RNA sequencing, total RNA was isolated from tissue samples by using miRVana RNA isolation kit (ThermoFisher Scientific, Waltham, MA) following the total RNA procedure. RNA was eluted using RNase-free water and stored at − 80 °C. RNA from serum was isolated using the Qiagen Plasma/Serum kit (Qiagen, Hilden, Germany) using 200 μL of serum.

For the miRNA and mRNA microarray experiments, total RNA was isolated with TRIzol reagent according to the manufacturer’s instructions (Invitrogen, Carlsbad, CA).

Exosomes were isolated from serum using size exclusion chromatography following the protocol of Böing et al. [32]. RNA was subsequently isolated using the above mentioned Qiagen Plasma/Serum kit.

### RNA quantification and quality assessment of isolated RNA

RNA purity and concentration was measured with NanoDrop™ ND-1000 spectrophotometer (Thermo Fisher Scientific, Waltham, MA). For further assessment of RNA quality and relative size, samples were measured using Eukaryote total RNA assay on the Agilent 2100 Bioanalyzer (Agilent Technologies, Santa Clara, CA) to detect the presence of small RNAs and to calculate RIN values. A RIN value > 9 was regarded as high quality and sufficient for sequencing.

### Preparation of cDNA library for small RNA sequencing

The tissue validation dataset was prepared using NEBNext® Small RNA Library Prep (#E7330L) and the serum validation data set was prepared using the TruSeq small RNA protocol (Illumina, San Diego, CA), both according to the manufacturer’s instructions. The paired tissue, serum and exosome samples for the discovery dataset and the validation dataset was prepared using NEXTFLEX® Small RNA-Seq Kit (#NOVA-5132-06), according to the manufacturer’s instructions. PCR Amplification was performed using 13 cycles. Calibrator RNAs were added during the 3′ ligation step as previously described [33, 34]. The miRNA fragments were sequenced on the Illumina HiSeq 2500 and HiSeq 4000 systems (Illumina, San Diego, CA) using 50 base pair single read, at the Genomics Core Facility (GCF) in Trondheim, Norway.

### Processing of sequence data

The raw data was processed according to Mjelle et al. [34] to identify mature miRNAs and isomiRs.

### MicroRNA microarray analysis

The miRNA experiment was performed in Center of Excellence in Translational Medicine on Fundeni Clinical Hospital using One-colour Microarray-based Gene Expression (Agilent Technologies, Santa Clara, CA, USA) according to manufacturer protocol.

Briefly, total RNA (100 ng) was dephosphorylated, labeled with Cyanine 3-pCp using miRNA Complete Labeling and Hyb Kit. The labeled miRNAs were hybridized on G3 Human miRNA Microarray Kit, Release 21, 8 × 60 K. After hybridization and washing, slides were scanned with Agilent Microarray Scanner with SureScan High -Resolution Technology (G2505B).

### Messenger RNA microarray analysis

Messenger RNA microarray was performed using the Agilent technology. Specifically, the samples were run using the protocol “One Color Microarray-Based Gene Expression Analysis, Low Input Quick Amp Labelling, version 6.9.1, August 2015”. Data were extracted from the machine using the software “Feature Extraction 12.0.3.1” and the microarray slide was scanned using “Agilent Microarray Scanner G250”.

The acquired data from miRNA and mRNA microarray was analyzed using Scan Control software and further extracted with Feature Extraction 12.0.3.1(Agilent Technologies) in order to obtain fluorescent image slides in TIF format and respectively raw data with signal intensity values.

### Differential expression analysis

Differentially expressed miRNAs and isomiRs were identified using the Bioconductor package limma combined with voom transformation. To compare miRNA expression between samples, read counts were normalized using counts per million (cpm) normalization. For miRNAs, ncRNAs and isomiRs, we required expression of 1 cpm in at least 50% of the samples when comparing tumor vs normal. The miRNA microarray data was analyzed in R using the Bioconductor package AgiMicroRna. The mRNA microarray data was analyzed in R using the Bioconductor package limma and the functions normalizeBetweenArrays and backgroundCorrect.

### Gene ontology analysis

Gene ontology analysis on mRNAs was performed using the Bioconductor package clusterProfiler and the enrichGO profiler with the parameters *p* value Cutoff = 0.01; pAdjustMethod = “BH”. Up-regulated and down-regulated transcripts were defined as transcripts with adjusted *p*-value below 0.05 and log2 fold change (logFC) above 0.5 or below − 0.5, respectively. The “universe” was defined as transcripts detected in at least 50% of the samples. Only probes that could be assigned a unique Entrez ID were included in the analysis. To reduce redundant GO terms we used the “simplify” function in clusterProfiler with default parameters.

### Gene ontology analysis for miRNA targets

Sixty-nine miRNAs were differentially expressed in both miRNA sequencing data and in microarray data. TargetScan gave us ~ 8500 unique target genes for these miRNAs. We further filtered the number of miRNA-target gene pairs based on expression levels of miRNA (keeping highly expressed miRNAs having 75% percentile expression across samples > 10 log_2_ cpm), weighted context scores from TargetScan (less than − 0.4) and selecting the miRNA target gene pairs, if more than 75% of the number of miRNAs targeting one gene are either all up or all downregulated [35]. Further, we selected only the negatively correlated miRNA-target gene pairs. We finally had 31 miRNA and 315 unique target genes. Gene Ontology (GO) enrichment analysis was done for target genes of both up and downregulated miRNAs with gProfileR package in R.

### Survival analysis

The survival analyses were performed in R by using the package “survival” and the survival-plots were generated using “ggsurvplot”. “High” and “Low”-expression in the plots indicate the 50% highest and lowest percentile of the expression values. The *p*-values were calculated form a coxph-model and were adjusted for multiple testing by using Bonferroni Correction.

## Results

### Overview of the study and data

Our study material consisted of liver tumor and adjacent normal liver samples from HCC patients, serum samples from the same patients and from non-cancer controls, and serum exosomes isolated from the patient serum samples (Additional file 1: Figure S1). We divided the participants into a discovery cohort, consisting of the 19 non-cancer controls and 17 patients (cases) with matched tissue, serum, and exosome samples, and a validation cohort, consisting of the remaining patients.

In the discovery cohort, we used sRNA-seq to characterize miRNAs and other small RNAs in the samples. The aim of this experiment was to identify candidate HCC biomarkers by investigating the distribution of small RNAs in the different biological sample types, identifying expression differences between tumor and normal tissue, patient and control serum, and patient serum and serum exosomes, and investigating correlations in expression between exosome, serum and tissue samples.

We then used the larger validation cohort to confirm the results from the discovery cohort comparisons and to identify RNAs that correlated with clinical parameters. Specifically, we did a second sRNA-seq experiment that included 80 serum samples, 8 matched exosome samples, 78 tumor tissue samples, 80 normal tissue samples (78 matched tumor and normal tissue samples). For additional validation, 73 of the matched tissue samples were analyzed on miRNA microarrays. Finally, we used mRNA microarrays to analyze 59 of the matched tissue samples to identify differentially expressed mRNAs, mRNAs correlating with clinical parameters, and target candidates for the differentially expressed miRNAs. The following sections detail our results.

### MicroRNA expression in tissue, serum and serum exosomes

To detect differentially expressed miRNAs between sample classes and to identify potential correlations between the circulating miRNAs and tissue miRNAs, we sequenced small RNAs in tumor and normal tissue, and whole serum and serum exosomes collected from 17 patients (discovery cohort; Additional file 2: Table S1). For comparison, we sequenced small RNAs in whole serum from 19 non-cancer controls. On average, 11,460,874 reads mapped to the human genome. Of these, on average 3,509,859 reads aligned to miRBase and 5,496,217 reads aligned to the RNACentral database of ncRNAs (see Methods) (Additional file 3: Figure S2A). The composition of detected RNAs varied across the sample types; exosome samples were depleted for transfer RNAs (tRNAs) compared to serum and tissue, whereas tissue samples were enriched for small nucleolar RNAs (snoRNAs) compared to serum and exosome samples (Additional file 3: Figure S2B). When analyzing the relative expression of the different ncRNAs within each sample type we observed high expression of small cytoplasmic RNAs (scRNAs), which included Y RNAs, in the serum and exosome samples and high expression of snoRNAs in the tissue samples (Additional file 3: Figure S2C). All samples included a set of 10 calibrator RNAs that were added during sample preparation before sequencing and used for normalization in all analysis (Additional file 4: Table S2).

**Fig. 1:**
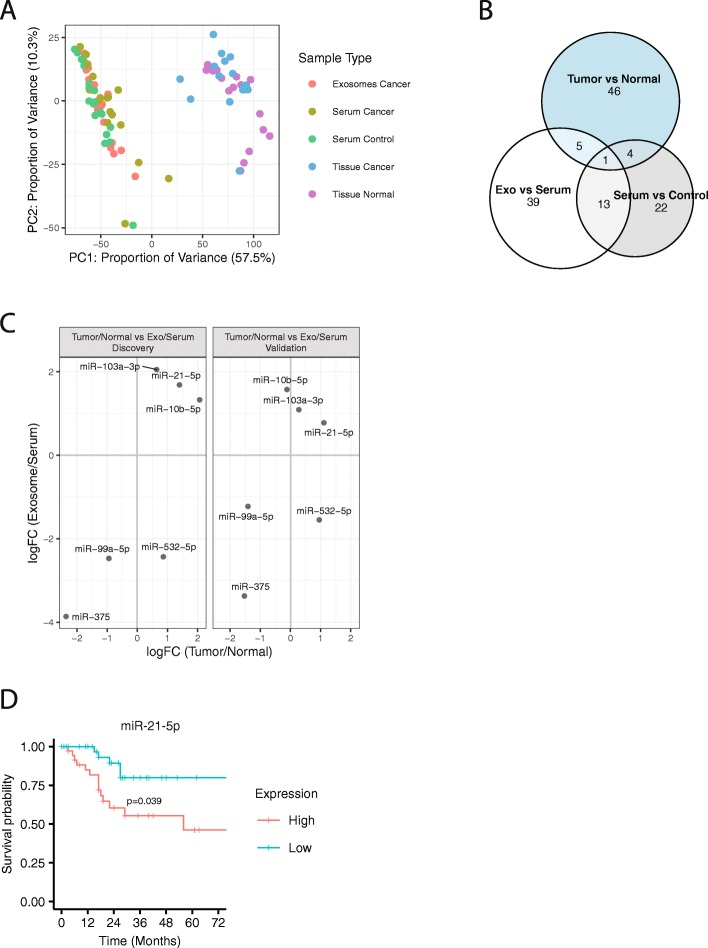
**a** PCA plot of mature miRNA expression in tissue, serum and exosomes samples in the discovery dataset. Dots represent samples and are colored according to sample type. **b** Venn diagram of differentially expressed miRNAs for the samples shown in A). Each circle represents a specific sample type comparison. **c** Comparison of miRNA logFC values for the exosomes vs serum (y-axis) and tumor tissue vs normal tissue (x-axis) comparisons in the discovery samples (left) and the validation samples (right). Shown are miRNAs that were significant in both comparisons (tumor vs normal and exosome vs serum) in the discovery samples. **d** Overall survival plot for patients in the validation dataset based on miR-21-5p expression in tumor tissue (*n* = 78). The y-axis shows overall survival probability and the x-axis shows survival time in months. “High” and “Low” represent miR-21-5p expression above and below the median, respectively

A principal component analysis (PCA) of mature miRNA expression found that the tissue samples were markedly different from the serum and exosome samples (Fig. 1a; PC1). Within the serum and exosome samples, control and cancer serum formed partially overlapping sub-clusters along the PCA’s second principal component (PC2), with the cancer exosome samples clustering between the two serum groups. In comparison, except for two cancer samples, the cancer and normal tissue samples formed nearly distinct clusters along PC2. Overall, the intra-group variation was lower for the tissue samples than for the serum and exosome samples, and normal tissue had the lowest intra-group variation in miRNA expression.

### Differentially expressed miRNAs, isomiRs, and ncRNAs in tissue, exosomes, and serum

To investigate specific expression differences between the sample types for the 17 patients and control samples, we performed differential expression analysis on tumor vs normal tissue, exosomes vs cancer serum, and cancer serum vs control serum. We detected 58 significant miRNAs between exosomes and cancer serum (Additional file 5: Figure S3A), 40 significant miRNAs between cancer serum and control serum (Additional file 5: Figure S3B), and 56 significant miRNAs between tumor and normal tissue (Additional file 5: Figure S3C, Additional file 6: Table S3). In general, we observed different sets of significant miRNAs between the three comparisons, of which tumor tissue vs normal tissue had the highest number of unique significant miRNAs (Fig. 1b). One miRNA, miR-532-5p, was significant across all three comparisons.

Next, we focused on isomiRs (Additional file 7: Figure S4A) and other ncRNAs that did not match any known miRNAs. We detected 176 significant isomiRs between exosomes and cancer serum, 237 significant isomiRs between tumor and normal tissue and 123 significant isomiRs between cancer serum and control serum (Additional file 7: Figure S4B). For the ncRNAs, we detected 20 significant ncRNAs between tumor and normal tissue, 64 significant ncRNAs between exosomes and cancer serum and 48 significant ncRNAs between cancer serum and control serum. We observed an enrichment of down-regulated snoRNAs in tumor tissue vs normal tissue (Additional file 7: Figure S4C). In exosome vs cancer serum, most of the highly significant ncRNAs were down-regulate in exosomes (Additional file 7: Figure S4D). In cancer serum vs controls serum, we observed an enrichment of up-regulated snoRNAs (Additional file 7: Figure S4E).

### Expression differences between tumor and normal tissue are partly mirrored between serum exosomes and whole serum

As tumor cells release miRNA-containing exosomes that enter circulation and can have both local and remote functions [36], we reasoned that tumor-associated miRNAs could be enriched in serum exosomes compared with whole serum. Of the 114 miRNAs that were significant in either the exosome vs cancer serum or tumor vs normal tissue comparison, 77 unique miRNAs were expressed in all four sample types and 37 miRNAs had identical signs of their log fold change (logFC) values between the two comparisons (Additional file 8: Figure S5A). Focusing on the six miRNAs that were significant in both comparisons (exosome vs cancer serum and tumor vs normal), five were either up-regulated (logFC> 0; 2 miRNAs) or down-regulated (logFC< 0; 3 miRNAs) in both tumors and exosomes compared with normal tissue and whole serum, respectively (Fig. 1c). The up-regulated miRNAs were the oncogenic miRNAs miR-21 and miR-10b, supporting that miRNA dysregulation in tumors is reflected in circulation.

To validate these results, we performed a second sequencing experiment that included eight patients with matched exosome and cancer serum samples and 75 patients with matched tumor and normal tissue samples of which five patients were matched across all four sample types (validation cohort; Additional file 2: Table S1). Because of the higher number of patients, we detected a higher number of miRNAs with significant expression differences between tumor and normal tissue when considering all 75 patients (Additional file 6: Table S3). All miRNAs that were significant between tumor and normal tissue in the discovery dataset showed consistent expression in the validation dataset, when using all 75 patients, except miR-183-3p and miR-34a-3p which were not detected in the validation dataset (Additional file 8: Figure S5B).

For the correlation analysis, we used data from the validation cohort patients that were matched across all four sample types. The results showed that five of the six miRNAs had similar direction of expression in this new cohort, with miR-21 being positively correlated in both cohorts (Fig. 1c).

As miR-21 reproducibly informed on tumor dysregulation within both tissue and serum samples, we next asked whether this miRNA could inform on patient survival. A survival analysis in the validation dataset found that high expression levels of miR-21 within tumor tissue was significantly associated with poorer survival (Fig. 1d). We found no significant association between patient survival and miRNA expression in serum or normal tissue.

Next, we investigated whether isomiRs and other ncRNAs also showed correlated expression differences between tumor and normal tissue and serum exosomes and whole serum. In the discovery cohort data, we detected 15 isomiRs that were significant in both exosome vs cancer serum and tumor vs normal; 13 of these isomiRs were either up-regulated or down-regulated in both tumors and exosomes compared with normal tissue and whole serum, respectively (Additional file 8: Figure S5C). In the validation cohort, 12 of these 15 isomiRs showed correlated expression differences, including isomiRs for miR-21, miR-122, miR-375 and other miRNAs.

Sixty-four ncRNAs were significant in exosome vs cancer serum, 20 ncRNAs were significant in tumor vs normal, and one ncRNA were significant in both comparisons. Most ncRNAs were up-regulated in tumor vs normal and downregulated in exosomes vs serum and only one of the significant ncRNAs were detected in the validation cohort.

In summary, the miRNA miR-21 showed increased mature and isomiR expression in tumor and serum exosomes compared with normal liver and cancer serum, supporting a model were tumor cells more than normal cells release miR-21-containing exosomes into circulation.

### Correlating miRNA expression in serum samples with clinical parameters

Based on the second sRNA-seq experiment on 75 patients we asked if the expression of miRNAs in serum correlated with the patients’ clinical parameters. A PCA plot showed that sample age is one of the main factors explaining the variation in miRNA expression, suggesting that RNA degradation may contribute significantly to miRNA expression profiles (Additional file 9: Figure S6A). All further analysis on the serum samples were therefore adjusted for sample age.

We detected two miRNA, miR-194-5p and miR-122-3p, as significantly upregulated in patients with HBV infection (Table 1). When comparing resection versus transplantation regimens we detected 17 miRNAs that were significantly differentially expressed between the two treatment groups (Table 1).

**Table 1:** Differentially expressed serum miRNA across clinical parameters

Comparison	miRNA	Fold change	Average expression	Adjusted *P*-value
Transplantation vs Resection				
	hsa-miR-651-5p	1.690	2.692	0.003
	hsa-miR-6511b-3p	−2.107	2.524	0.005
	hsa-miR-197-3p	−1.888	6.484	0.007
	hsa-miR-92b-3p	−1.571	8.678	0.018
	hsa-miR-374a-3p	1.801	3.822	0.022
	hsa-miR-6511a-3p	−2.094	2.999	0.022
	hsa-miR-7706	−1.036	5.388	0.022
	hsa-miR-362-3p	1.457	1.880	0.023
	hsa-miR-483-3p	−2.412	2.966	0.026
	hsa-miR-3605-3p	−1.487	5.241	0.034
	hsa-miR-125a-5p	−1.962	8.930	0.042
	hsa-miR-425-5p	−0.972	10.199	0.046
	hsa-miR-450b-5p	1.554	6.006	0.046
	hsa-miR-205-5p	−2.338	2.917	0.046
	hsa-miR-6741-3p	−1.978	1.678	0.046
	hsa-miR-378c	1.312	6.059	0.046
	hsa-miR-550a-3p	−1.893	4.457	0.046
HBV vs Non-HBV				
	hsa-miR-122-3p	2.929	2.364	8.45E-05
	hsa-miR-194-5p	1.470	7.536	0.007

“Comparison” shows the clinical parameters between which the comparison was performed; “miRNA” shows the miRBase miRNA name; “Fold Change” shows the log2 fold change value for the miRNA; “Average Expression” shows the average expression (cpm, log2) of the miRNA across samples; “Adjusted *P*-value” shows the Benjamini-Hochberg asdjusted *p*-value. Positive fold change indicates upregulation in transplantation or HBV for the two comparisons respectively

### MicroRNA expression in tissue correlates with surgery type, HBV infection, and cirrhosis

Having analyzed the serum samples, we went on investigating miRNA expression in tumor and normal tissue for the same patients. The tissue samples were analyzed using both sequencing and microarray approaches. First, we investigated the correlation between the two platforms by comparing fold-change values for the differences between tumor and normal. We detected 137 significant miRNAs using microarrays and 234 significant miRNAs using sequencing; 68 miRNAs were significant on both platforms (Additional file 6: Table S3). We observed high correlation between the platforms and when focusing on miRNAs that were detected on both platforms and significant on at least one of the platforms, 116 of 128 miRNAs (91%) were changing in the same direction (Additional file 9: Figure S6B). Similar as in the serum samples, we identified sample age as a major factor affecting miRNA expression profiles in the sequencing data (Additional file 9: Figure S6C-D). The microarray data, however, were not affected by sample age (Additional file 9: Figure S6E-F).

Further, we compared tissue miRNA expression with the clinical parameters, using the microarray data as a validation for the sequencing data. Comparing resection vs transplantation and adjusting for sample age, we identified 8 significant miRNAs in the tumor samples and 18 significant miRNAs in the normal samples (Table 2). For the tumor samples, six of the eight miRNAs were expressed in the same direction in the microarray; however, none were significant in the microarray. For the normal samples, five of the 18 significant miRNAs were expressed in the same direction in the microarray; however, none were significant in the microarray.

**Table 2:** Differentially expressed tissue miRNAs across clinical parameters as measured by sequencing and microarray

Comparison	miRNA	Fold change sequencing	Adjusted *P*-value sequencing	Fold change microarray	Adjusted *P*-value microarray
Transplantation vs Resection in Tumor Tissue					
	hsa-miR-107	−0.902	0.041	−0.317	0.844
	hsa-miR-17-5p	−1.179	0.041	−0.439	0.844
	hsa-miR-339-5p	−1.255	0.041	NA	NA
	hsa-miR-505-3p	−1.077	0.042	−0.170	0.966
	hsa-miR-20a-5p	−1.150	0.043	−0.364	0.844
	hsa-miR-532-3p	−1.276	0.043	−1.157	0.841
	hsa-miR-1248	−1.847	0.049	NA	NA
	hsa-miR-93-5p	−0.949	0.049	−0.168	0.941
Transplantation vs Resection in Normal Tissue					
	hsa-miR-224-5p	1.328	0.005	0.689	0.584
	hsa-miR-451a	−1.977	0.005	0.205	0.817
	hsa-miR-193b-3p	−1.202	0.011	0.367	0.539
	hsa-miR-19b-3p	−0.972	0.015	−0.114	0.821
	hsa-miR-16-5p	−0.989	0.015	−0.023	0.951
	hsa-miR-543	1.451	0.028	NA	NA
	hsa-miR-345-5p	−0.767	0.028	NA	NA
	hsa-miR-324-5p	−1.354	0.028	0.581	0.410
	hsa-miR-101-5p	−1.298	0.030	NA	NA
	hsa-miR-17-3p	−0.922	0.030	0.412	0.582
	hsa-miR-675-3p	−1.374	0.032	NA	NA
	hsa-miR-485-3p	1.206	0.032	NA	NA
	hsa-miR-128-3p	1.087	0.032	0.504	0.535
	hsa-miR-195-3p	0.887	0.032	NA	NA
	hsa-miR-146b-5p	0.710	0.038	0.420	0.572
	hsa-miR-15b-3p	−1.170	0.038	NA	NA
	hsa-miR-652-3p	−1.019	0.038	0.664	0.503
	hsa-miR-660-3p	−1.126	0.047	NA	NA
Cirrhosis vs Non-Cirrhosis in Normal Tissue					
	hsa-miR-146b-5p	1.027	0.003	1.128	0.031
Cirrhosis vs Non-Cirrhosis in Tumor Tissue					
	hsa-miR-141-3p	−2.886	0.003	NA	NA

“Comparison” shows the clinical parameters between which the comparison was performed; “miRNA” shows the miRBase miRNA name; “Fold Change Sequencing” shows the log2 fold change value for the miRNA in the sequencing data; “Adjusted *P*-value Sequencing” shows the Benjamini-Hochberg asdjusted *p*-value in the sequencing data. “Fold Change Microarray” shows the log2 fold change value for the miRNA in the microarray data; “Adjusted *P*-value Microarray” shows the Benjamini-Hochberg asdjusted *p*-value in the microarray data. “NA” indicates that the miRNA is not detected. Positive fold change indicates upregulation in transplantation or cirrhosis for the two comparisons

Comparing cirrhosis and non-cirrhosis, we identified miR-141-3p to have lower expression in the tumor samples of cirrhotic as compared with non-cirrhotic patients and miR-146b-5p to have higher expression in the normal samples of cirrhotic compared with non-cirrhotic patients (Table 2). The microarray data confirmed the expression differences for miR-146b-5p, whereas miR-141-3p was not detected as expressed in the microarray data.

### Gene expression in tissue correlates with surgery type, HBV infection and cirrhosis

Next, we used microarrays to measure gene expression profiles in tumor and normal samples from 59 HCC patients. Of the 50,739 measured probes we detected 6280 differentially expressed transcripts between tumor and normal samples. Focusing on genes with an average log_2_ expression signal above 10, we observed a trend towards more down-regulated genes in tumor compared to normal, especially for the most significant genes (Additional file 10: Figure S7A). A PCA plot revealed clustering of the normal samples and more distributed tumor samples, indicating tumor samples to be more heterogeneous than normal samples (Additional file 10: Figure S7B). We observed no clustering related to the main clinical parameters (Additional file 10: Figure S7B). We performed gene ontology (GO) analysis on the significantly up-regulated and down-regulated transcripts (see Methods). The GO analysis showed that genes up-regulated in tumors were enriched for terms related to cell division and DNA replication, whereas genes down-regulated in tumors were enriched for terms related to metabolism and lipid (Additional file 11: Figure S8A). Subsequent KEGG analysis on the same two groups revealed enrichment of the terms cell cycle and DNA replication for the up-regulated genes and metabolism for the down-regulated genes (Additional file 11: Figure S8B). In addition, for the up-regulated genes we found enrichment of the terms alcoholism and viral carcinogenesis, two factors that are considered as major risk-factors in HCC.

Next, we compared gene expression with clinical parameters and detected significant genes with respect to cirrhosis, HBV, and treatment. Specifically, we detected 14 significant genes between cirrhosis and non-cirrhosis for the tumor samples; five significant genes between resection and transplantation for the normal samples and 13 significant genes between HBV and non-HBV for the normal samples (Table 3).

**Table 3:** Differentially expressed genes across clinical parameters

Comparison	Official gene name^a^	Ensemble gene name	Fold change	Adjusted *P*-value
HBV vs Non-HBV in Normal Tissue				
	KANK4	ENSG00000132854	−1.798	0.021
	GRHL1	ENSG00000134317	−0.618	0.021
	lnc-C2orf54–2:1	NA	−1.626	0.034
	RRN3P2	ENSG00000103472	0.785	0.034
	ST13	ENSG00000100380	−0.603	0.044
	USP41	ENSG00000161133	−0.753	0.047
	lnc-METTL4–1:1	NA	0.358	0.047
	PATZ1	ENSG00000100105	−0.557	0.047
	NA	ENSG00000274021	−0.508	0.048
	lnc-C1orf222–1:1	NA	−0.478	0.048
	SPDYC	ENSG00000204710	−0.443	0.048
	PTGER4P2-CDK2AP2P2	ENSG00000275450	−0.505	0.048
	AK126423	ENSG00000278934	−0.441	0.048
Cirrhosis vs Non-Cirrhosis in Tumor Tissue				
	HOXB13	ENSG00000159184	−2.198	0.011
	NAP1L6	ENSG00000204118	−1.501	0.011
	EIG121	ENSG00000116299	−1.461	0.013
	CSMD3	ENSG00000164796	−0.598	0.024
	VPS9D1	ENSG00000261373	−0.822	0.024
	KIAA1324	ENSG00000116299	−1.250	0.031
	KCNMB2	ENSG00000197584	−0.617	0.037
	LOC101929022	NA	−0.595	0.037
	TCP1	ENSG00000120438	−0.751	0.037
	MB	ENSG00000198125	−0.991	0.037
	TTLL6	ENSG00000170703	−0.697	0.038
	AXDND1	ENSG00000162779	−0.722	0.038
	lnc-CCRN4L-7:1	NA	−0.885	0.038
	PLCH1	ENSG00000114805	−0.413	0.044
Transplantation vs Resection in Normal Tissue				
	AL355096	ENSG00000258460	−1.059	0.004
	FGF23	ENSG00000118972	2.370	0.004
	TMED11P	ENSG00000215367	−0.430	0.016
	TCONS_l2_00028727	NA	0.685	0.034
	MT1H	ENSG00000205358	−3.002	0.038

^a^long non-coding RNAs use names from lncipedia.org

“Comparison” shows the clinical parameters between which the comparison was performed; “Official Gene Name” shows the official gene symbol; “Ensemble Gene Name” shows the Ensemble gene name; “Fold Change” shows the log2 fold change value for the gene; “Adjusted *P*-value” shows the Benjamini-Hochberg asdjusted *p*-value. “NA” indicates that the name is not available. Positive fold change indicates upregulation in HBV, Cirrhosis or Transplantation for the three different comparisons

Finally, we validated the gene expression differences between tumor and normal samples by analyzing a publicly available HCC dataset. We analyzed the dataset of Grinchuk et al. that contained 115 primary tumor samples and 52 adjacent normal tissue samples [37]. A correlation analysis between the two datasets showed that the logFC values for the comparison tumor vs normal were highly comparable (*r* = 0.56) (Additional file 12: Figure S9). Two thousand three hundred eighty genes were significant in both datasets and the correlation value was 0.76 when only including these genes. These results indicate that the differences in gene expression observed in the current study is highly comparable with other studies from different cohorts.

### Gene ontology analyses for miRNA target genes

Having established that both miRNAs and mRNAs are highly dysregulated in HCC tissue, we wanted to investigate if the differentially expressed miRNAs were targeting a specific set of mRNAs. We used 69 miRNAs that were differentially expressed in tumor vs normal tissue and identified their candidate target mRNAs using TargetScan [38] and additional filtering (see Methods). Gene ontology analysis showed that miRNAs down-regulated in tumor were targeting genes that were enriched for functions related to collagen, whereas miRNAs up-regulated in tumor were targeting genes that were enriched for functions related to regulation of neutrophils, legionellosis, and basal cell carcinoma (Additional file 13: Figure S10).

## Discussion

We here performed paired small RNA profiling of tumor and normal tissue and circulating exosomes and serum from HCC patients. Comparing the differences between tumor and normal tissue and the differences between exosomes and serum we detected six miRNAs that differed in both comparisons. Four of the six miRNAs were concomitantly enriched (two miRNAs) or depleted (two miRNAs) within tumor tissue and serum exosomes in both the discovery and validation data. The most striking is miR-21, which is upregulated in tumor tissue and in serum exosomes as well as being associated with overall survival. These findings indicate that the expression levels in serum exosomes of some miRNAs, including miR-21, depend on exosomes released from tumor cells. Indeed, increased exosomal miR-21 levels has been associated with cancer in other studies. In ovarian cancer, colon cancer, and lung cancer, positive correlations were found between miR-21 in the tumor and circulating miR-21 in exosomes [39–41]. It has been suggested that exosomal miR-21 could be used as a universal biomarker for cancer in combination with other clinical parameters [42]. In HCC, one study has shown a positive correlation between exosomal miR-21 in cell lines and miR-21 in the cell culture supernatant [43]. Another study showed significantly higher miR-21 levels in exosomes than in exosome-depleted serum or the whole serum [6]. Together, several previous results show that exosomal miR-21 could be a good biomarker for HCC, supporting our findings on patients’ samples.

Comparing miRNA expression in exosomes and whole serum we observed enrichment of several let-7 miRNAs in the exosomes. One study on gastric cancer cell lines showed that let-7 was secreted from cells via exosomes into the extracellular environment and thereby maintain the oncogenic properties of the cells [44]. Potentially more relevant to our results is the finding by Okoye et al. showing that Foxp3+ T regulatory (Treg) cells released exosomes containing let-7, and that these exosomes were further transferred to T helper 1 (Th1) cells, suppressing Th1 cell proliferation. This means that the increased let-7d levels we observed could be due to immune responses that cause exosomal let-7d, and potentially other let-7 members, to be released into circulation [45].

Comparing miRNA expression in serum from cancer patients and controls we observed a strong and highly significant upregulation of miR-122. MicroRNA miR-122 is highly expressed and strongly enriched in hepatocytes and is frequently downregulated in HCC tissue [46], which is shown to result in metastatic properties of hepatocytes [47]. Indeed, we found reduced levels of miR-122 in tumor tissue in our discovery set (*p*-value = 0.059), and a significant reduction in the validation set and in the microarray data (Additional file 6: Table S3). Reports also show that miR-122 is increased in serum of HCC patients [48, 49], in line with our results. In addition to increased level of miR-122, we generally observed a global upregulation of miRNAs in serum of cancer patients. Also, the miRNAs that were upregulated were affected to a much larger extent than the miRNAs that were downregulated.

Focusing on other small ncRNAs, we observed several significant ncRNAs in the different comparisons. In tissue, we observed a general downregulation of snoRNAs in tumor compared to normal. Studies have shown that snoRNAs are indeed downregulated in tumors [50] and in HCC [51]. In cancer serum vs control serum, snoRNAs were generally up-regulated and tRNAs were generally downregulated. In exosomes vs serum, we observed a similar trend as for miRNAs where most of the significant ncRNAs were downregulated in exosomes compared to serum.

A general theme for the sequencing data was that sample age was a confounding factor in the data, both in serum and in tissues. We observed separate clustering of samples collected recently compared to samples collected several years ago. Interestingly, the miRNA microarray data did not show similar clustering, indicating that the signal from the microarray probes could be less sensitive to RNA degradation or factors related to the samples’ age. In the tissue samples, the sample age bias is less evident than in the tumor samples, but is clearly visible in the normal samples. Notably, sample age largely overlaps with treatment type (resection vs transplantation), meaning that we cannot accurately predict if the bias is due to treatment or sample age. However, no clear separation with regard to treatment is found in the microarray data or in the tumor tissue.

Analyzing the tissue mRNA data, we found a large set of differentially expressed transcripts. The most significant of these mRNAs were regulated in tumor compared to normal tissue. Gene ontology analysis of the differentially expressed mRNAs revealed that cell cycle terms were enriched among genes upregulated in tumors. This is expected, as cell cycle genes often are dysregulated in HCC and cancer in general [52]. Downregulated genes were enriched for terms related to metabolism in general and lipid processes in particular. The liver plays an important role in catabolism of plasma lipoproteins and these processes are often impaired in liver cancer [53]. We also observed downregulation of some inflammation and complement related genes including CXCL14 and CFP. The chemokine CXCL14 is a known tumor suppressor involved in antimicrobial immunity and inflammatory processes [54]. Enriched KEGG pathways show similar biological functions as the gene ontologies. Here, alcoholism is enriched among the upregulated genes, in addition to cell cycle, microRNAs, and viral carcinogenesis. Alcohol and viral infections, especially hepatitis B and hepatitis C virus, are among the most significant risk factors of HCC. When comparing our gene expression results with the dataset of Grinchuk et al., the significant genes were generally changing in the same direction in the two datasets. Interestingly, and in accordance with the results above, the most significantly down-regulated genes common in both datasets were related to immunity. For instance, CLEC1B and CLEC4G are involved in T-cell immune responses and FCN2 is a liver-specific gene related to complement activation.

Finally, we investigated the gene ontologies for the predicted target genes of the dysregulated mRNAs in tissue. Here, several terms related to collagen were enriched among the mRNAs being targeted by miRNAs that were downregulated in tumor. This means that these genes have potentially less miRNA regulation and thereby increased expression in HCC tumors. Indeed, studies have shown that collagen related genes are upregulated in HCC and contribute to cancer progression [55, 56].

## Conclusions

This study indicates a correlation in miRNA and isomiR expression between exosomes and tissue, suggesting that these RNAs, including miR-21, are exported from tumors into circulation via exosomes. Further, the study presents a comprehensive profiling of miRNAs, other small ncRNAs, and mRNAs in HCC. Several of these RNAs are associated with clinical parameters such as Cirrhosis and HBV infection. We further found that high tumor expression of miR-21 is associated with poorer survival, pointing towards miR-21 as a potential prognostic and diagnostic biomarker for HCC. Gene ontology analyses of altered miRNA and mRNA expression pointed towards impaired lipid metabolism, increased cell proliferation and a miRNA-driven increase in collagen synthesis in HCC.

## Supplementary information

**Additional file 1:** **Figure S1.** Study overview.

**Additional file 2: Table S1.** Patient sample overview.

**Additional file 3: Figure S2.** Sequencing statistics.

**Additional file 4: Table S2.** Calibrator RNAs.

**Additional file 5: Figure S3.** Volcano plots miRNAs.

**Additional file 6: Table S3.** Differentially expressed miRNAs in tissue.

**Additional file 7: Figure S4.** IsomiRs and other ncRNAs.

**Additional file 8: Figure S5.** Correlation between exosomes and tissue.

**Additional file 9: Figure S6.** PCA plot of miRNAs.

**Additional file 10: Figure S7.** Volcano and PCA plot of mRNA data.

**Additional file 11: Figure S8.** GO plots of mRNA data.

**Additional file 12: Figure S9.** Correlation of mRNAs in Mjelle et al. and Grinchuk et al.

**Additional file 13: Figure S10.** GO plot of miRNA target data.

## Data Availability

All the data obtained and materials analyzed in this research are available with the corresponding author upon reasonable request.

## Abbreviations

Ago: Argonaute
CPM: Counts per million
DNA: Deoxy-ribonucleic acid
GO: Gene Ontology
HBV: Hepatitis B virus
HCC: Hepatocellular carcinoma
HCV: Hepatitis C virus
KEGG: Kyoto Encyclopedia of Genes and Genomes
logFC: Log fold change
miRNA: MicroRNA
miRs: MicroRNA
mRNA: Messenger RNA
ncRNAs: Non-coding RNA
OR: odds ratio
PC: Principal component
PCA: Principal component analysis
r: Pearson correlation coefficient
RFA: *Radiofrequency ablation*
RNA: Ribonucleic acid
scRNAs: Small cytoplasmic RNAs
snoRNAs: Small nucleolar RNA
sRNA-seq: Small RNA sequencing
Th1: T helper 1
tRNA: Transfer RNA
