# Supplementary figures and images for: Comprehensive transcriptomic analyses of tissue, serum, and serum exosomes from hepatocellular carcinoma patients
Source: BMC Cancer. 2019 Oct 28;19:1007. doi: 10.1186/s12885-019-6249-1 (PMC6816220; doi:10.1186/s12885-019-6249-1)

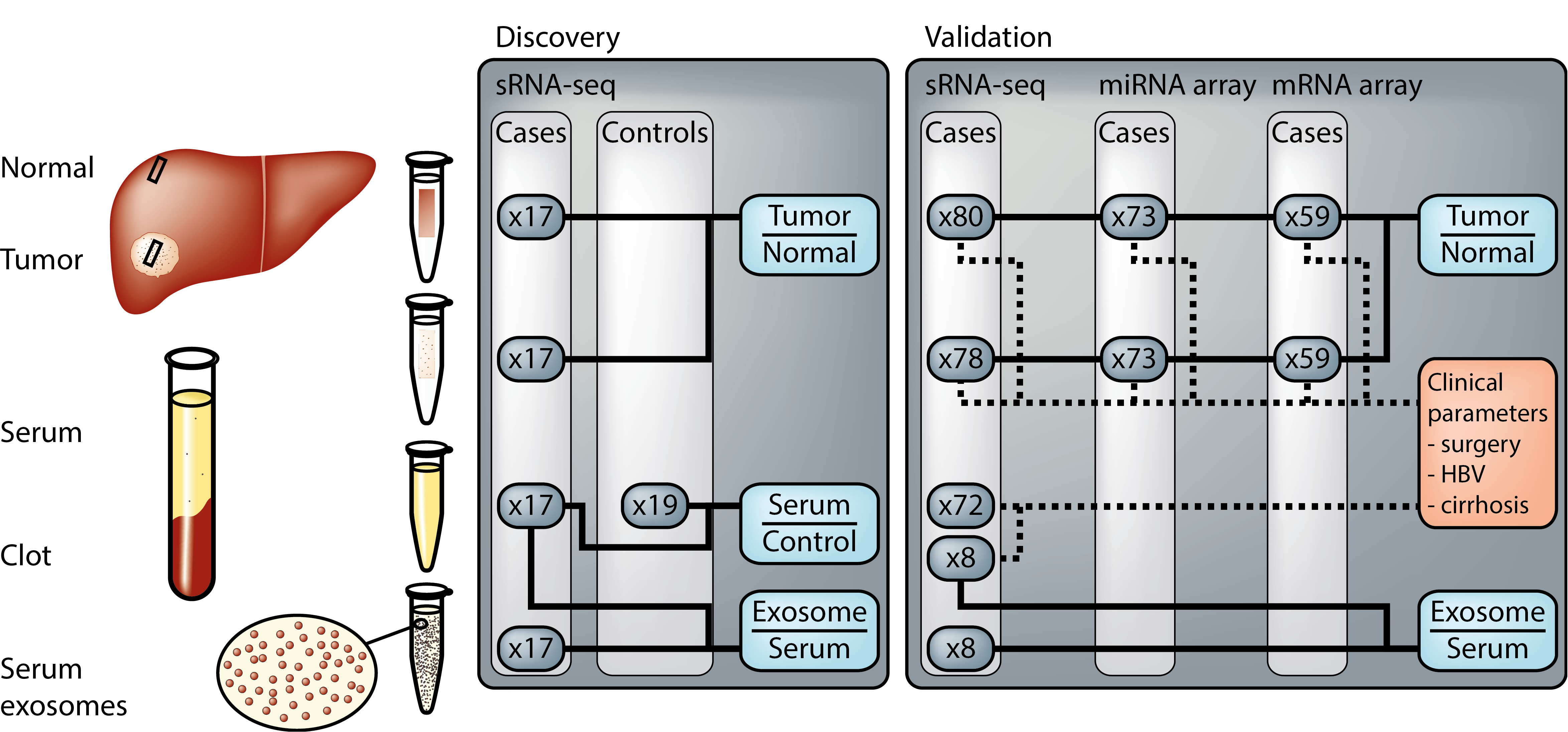

Supplement: Supplementary file 1 — Additional file 1: Figure S1. Study overview. [file 12885_2019_6249_MOESM1_ESM.png]

**A**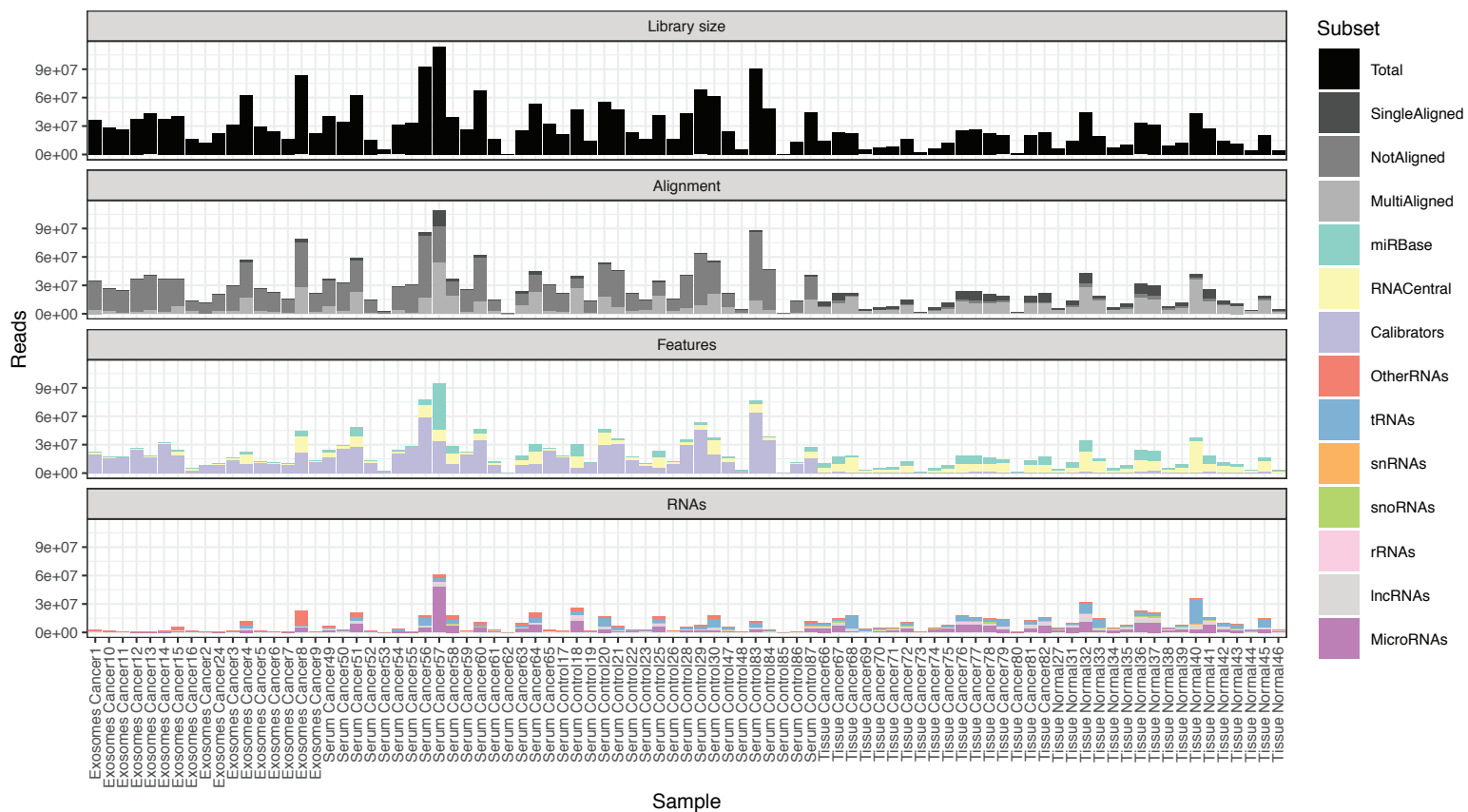**B**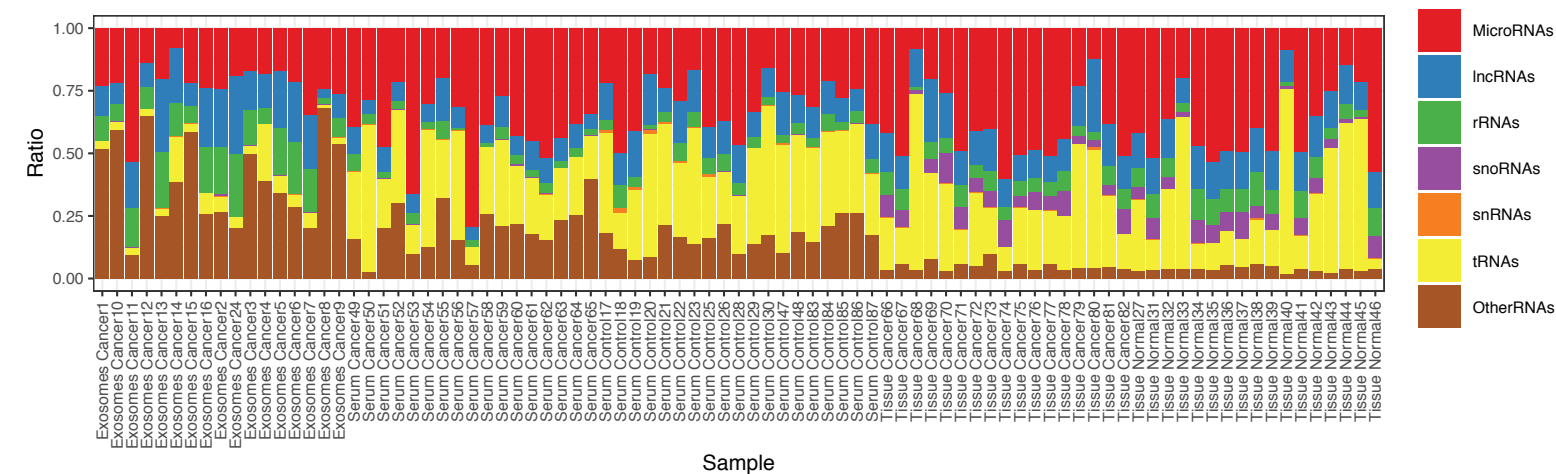**C**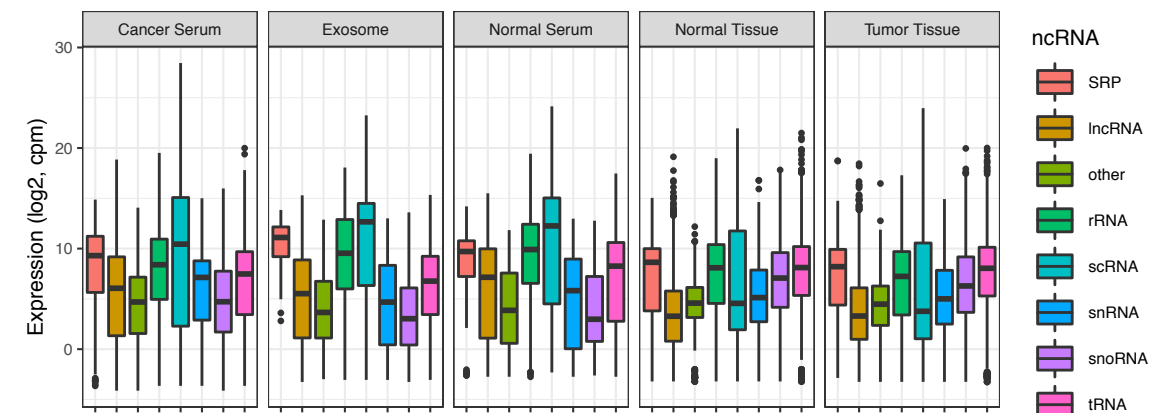

Supplement: Supplementary file 3 — Additional file 3: Figure S2. Sequencing statistics. [file 12885_2019_6249_MOESM3_ESM.pdf]

A

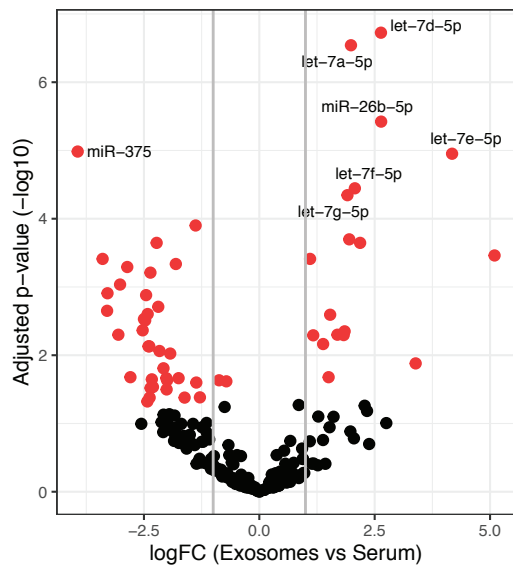

B

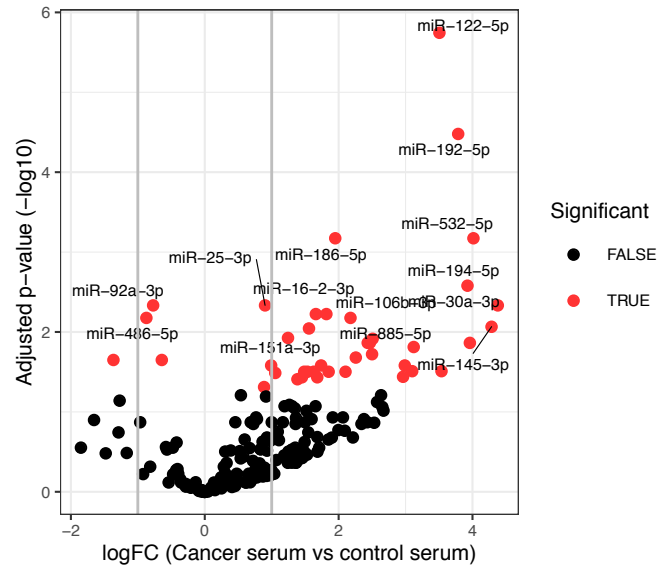

C

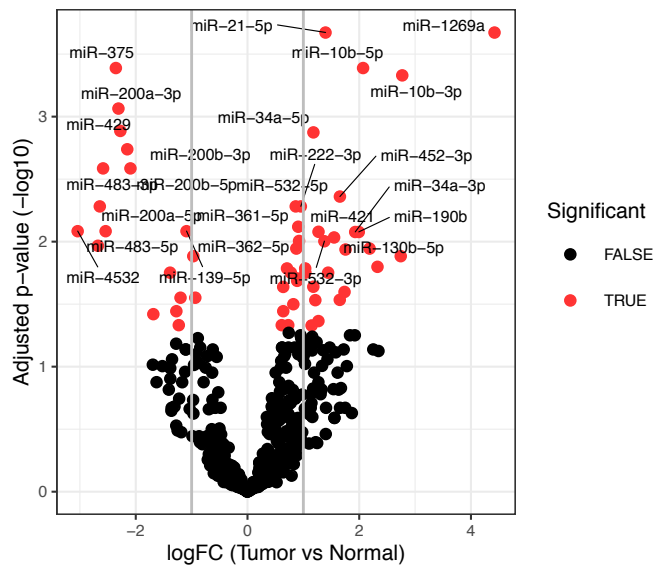

Supplement: Supplementary file 5 — Additional file 5: Figure S3. Volcano plots miRNAs. [file 12885_2019_6249_MOESM5_ESM.pdf]

A

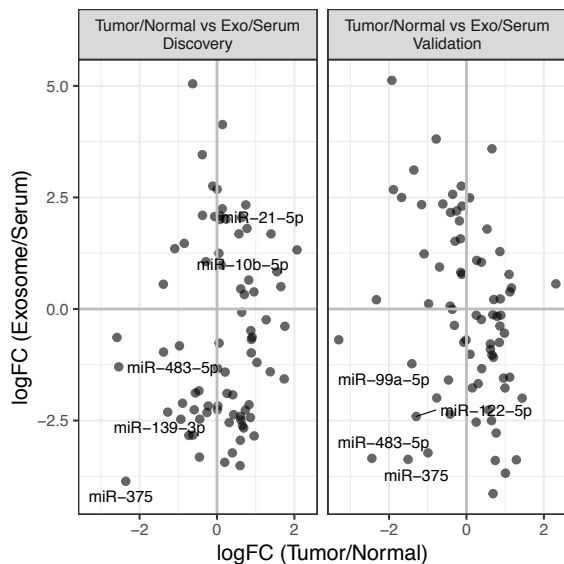

B

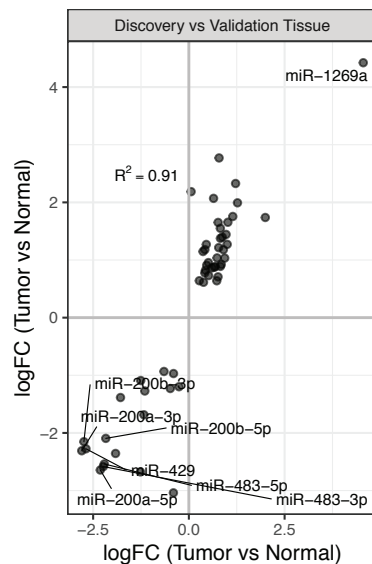

C

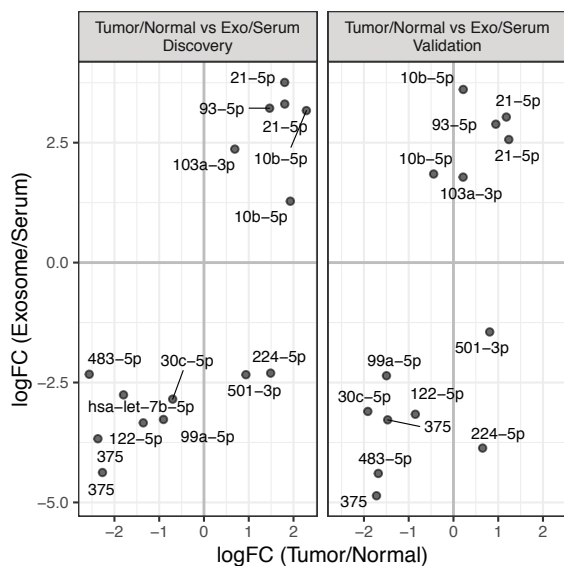

Supplement: Supplementary file 8 — Additional file 8: Figure S5. Correlation between exosomes and tissue. [file 12885_2019_6249_MOESM8_ESM.pdf]

A

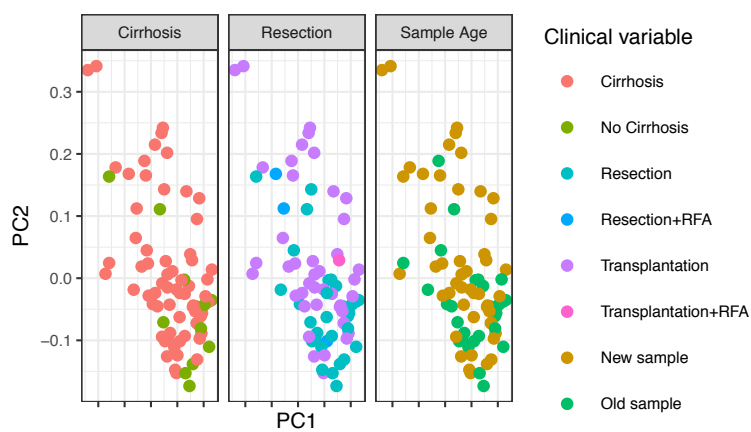

B

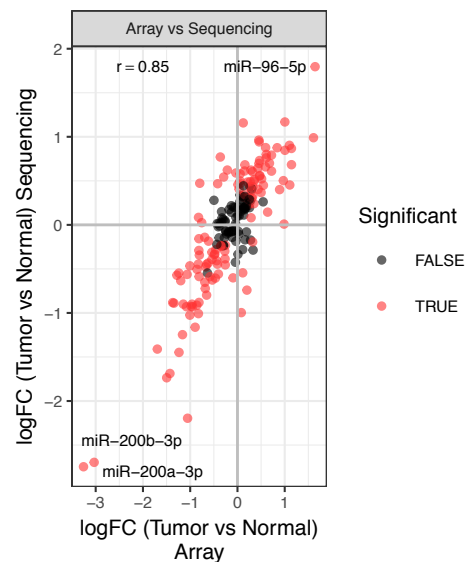

C

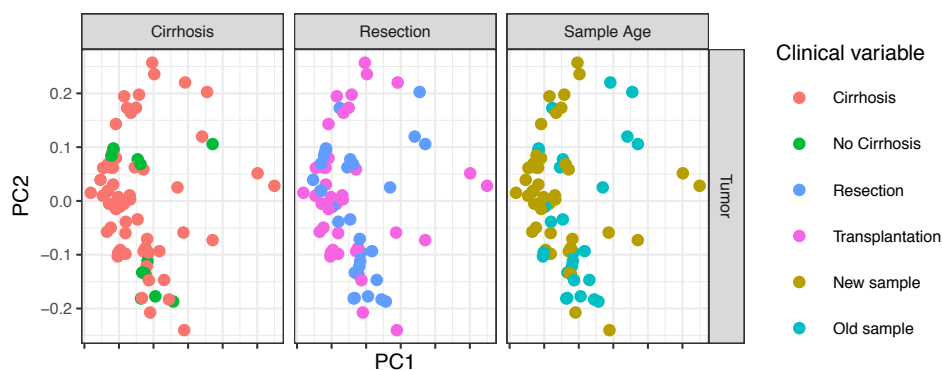

D

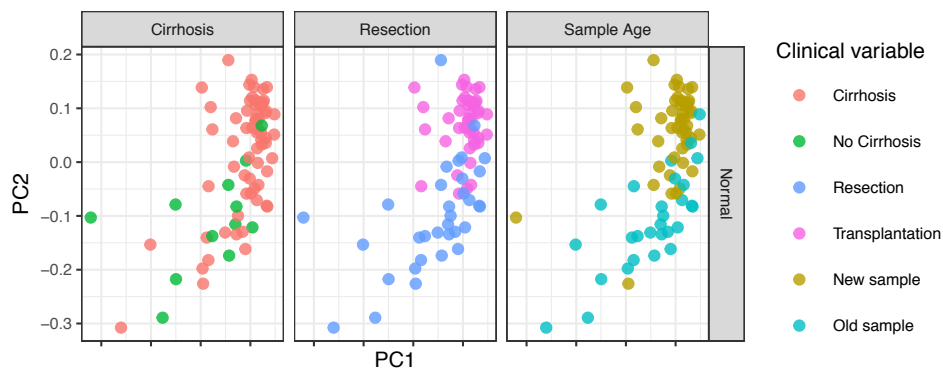

E

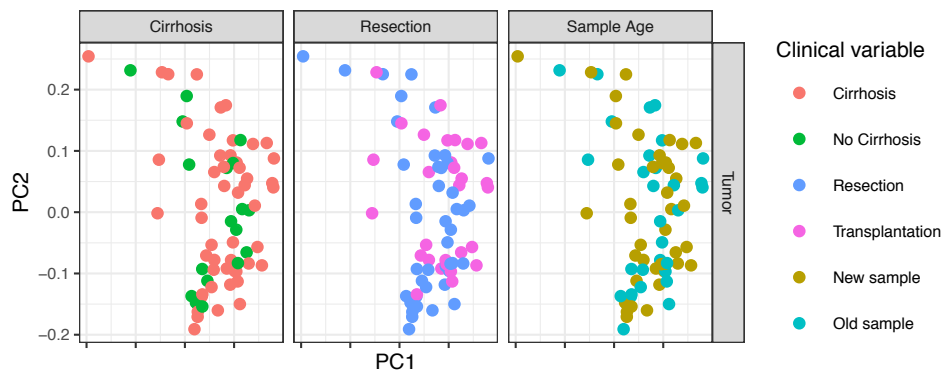

F

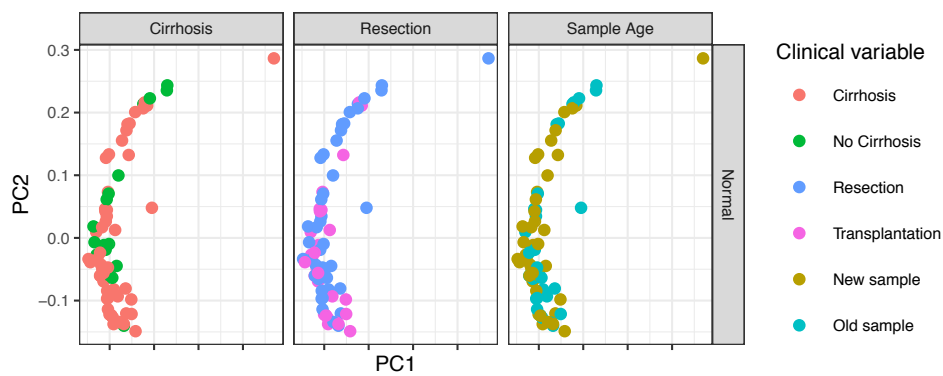

Supplement: Supplementary file 9 — Additional file 9: Figure S6. PCA plot of miRNAs. [file 12885_2019_6249_MOESM9_ESM.pdf]

A

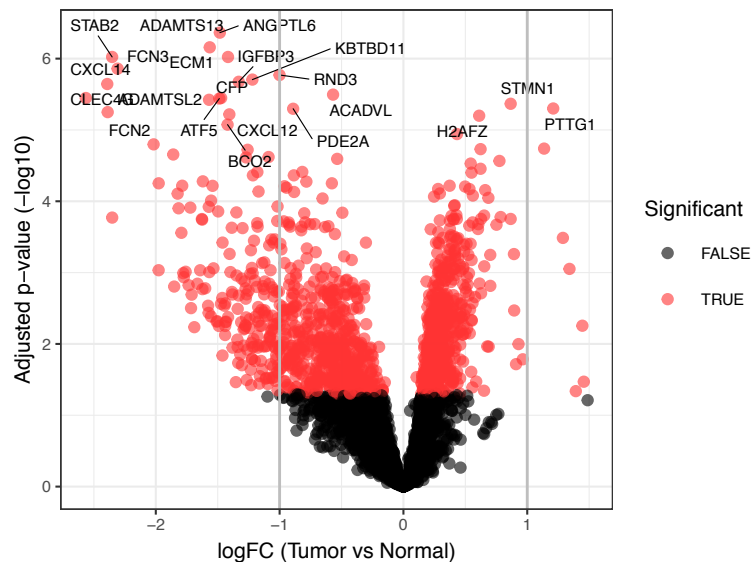

B

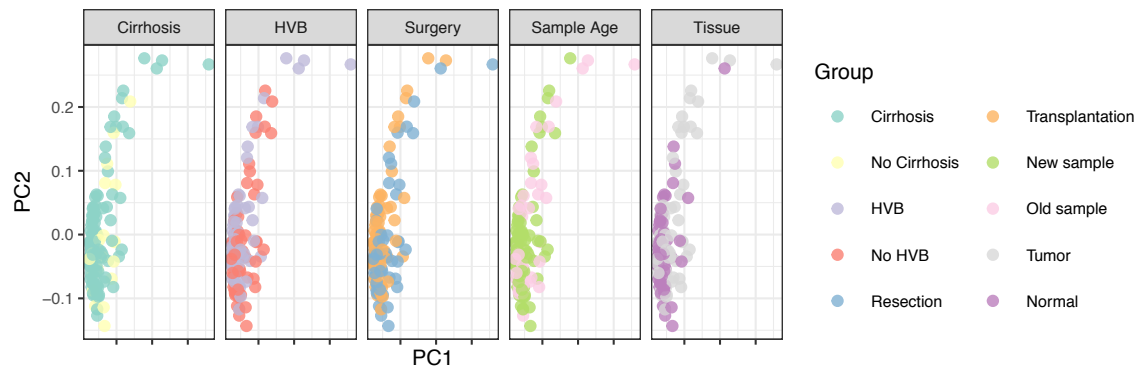

Supplement: Supplementary file 10 — Additional file 10: Figure S7. Volcano and PCA plot of mRNA data. [file 12885_2019_6249_MOESM10_ESM.pdf]

A

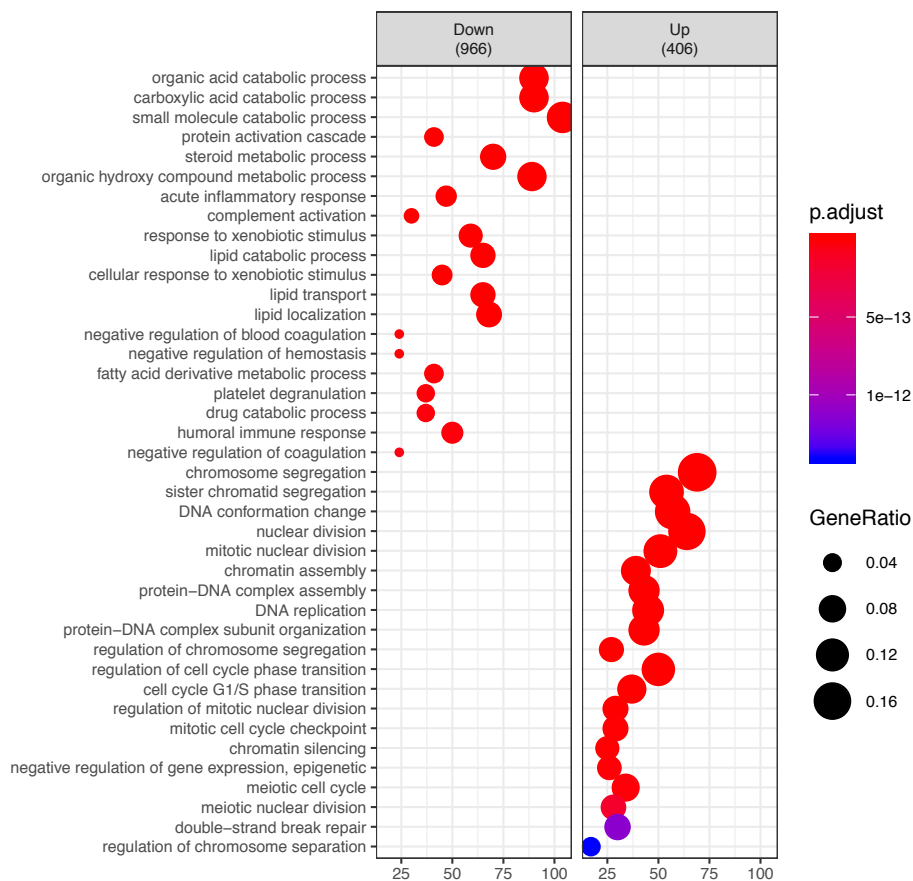

B

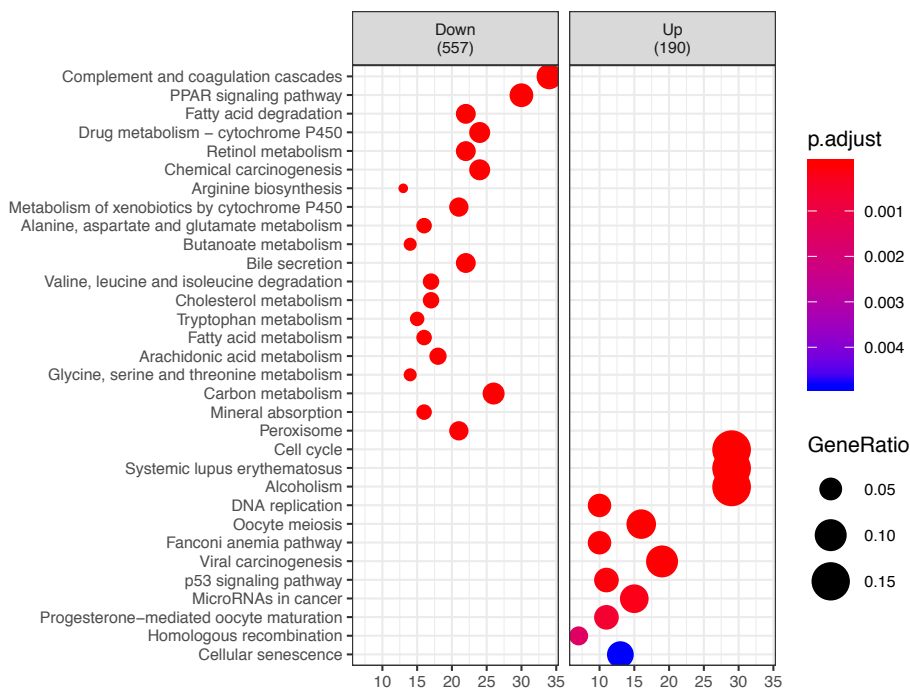

Supplement: Supplementary file 11 — Additional file 11: Figure S8. GO plots of mRNA data. [file 12885_2019_6249_MOESM11_ESM.pdf]

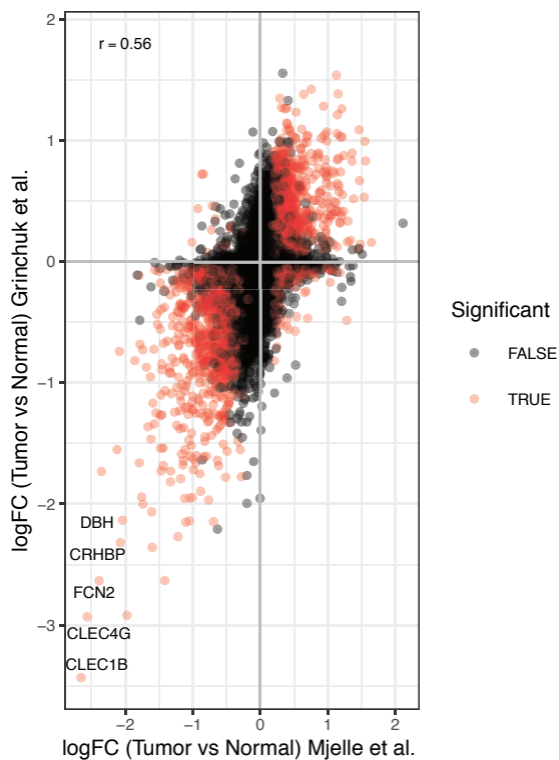

Supplement: Supplementary file 12 — Additional file 12: Figure S9. Correlation of mRNAs in Mjelle et al. and Grinchuk et al. [file 12885_2019_6249_MOESM12_ESM.pdf]
