# Supplementary material for: Comprehensive transcriptomic analyses of tissue, serum, and serum exosomes from hepatocellular carcinoma patients
Source: BMC Cancer. 2019 Oct 28;19:1007. doi: 10.1186/s12885-019-6249-1 (PMC6816220; doi:10.1186/s12885-019-6249-1)
Supplement: Supplementary file 7 — Additional file 7: Figure S4. IsomiRs and other ncRNAs. [file 12885_2019_6249_MOESM7_ESM.pdf]

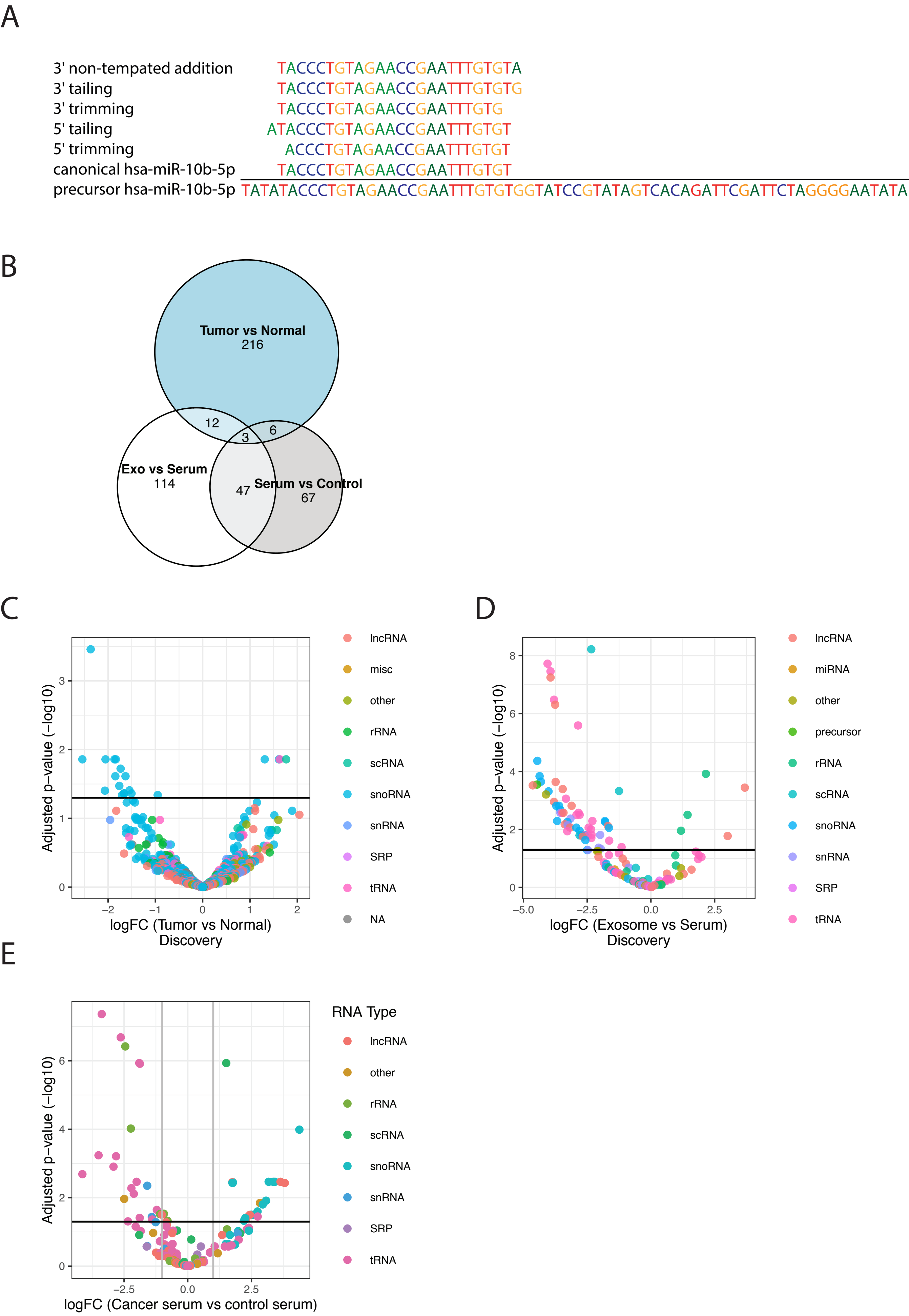
A
3' non-tempated addition TACCCTGTAGAACCGAATTTGTGTA
3' tailing TACCCTGTAGAACCGAATTTGTGTG
3' trimming TACCCTGTAGAACCGAATTTGTG
5' tailing ATACCCTGTAGAACCGAATTTGTGT
5' trimming ACCCTGTAGAACCGAATTTGTGT
canonical hsa-miR-10b-5p TACCCTGTAGAACCGAATTTGTGT
precursor hsa-miR-10b-5p TATATACCCTGTAGAACCGAATTTGTGTGGTATCCGTATAGTCACAGATTCGATTCTAGGGGAATATA
B
Tumor vs Normal
216
12
3
6
Exo vs Serum
114
47
Serum vs Control
67
C
Adjusted p−value (−log10)
logFC (Tumor vs Normal)
Discovery
lncRNA
misc
other
rRNA
scRNA
snoRNA
snRNA
SRP
tRNA
NA
D
Adjusted p−value (−log10)
logFC (Exosome vs Serum)
Discovery
lncRNA
miRNA
other
precursor
rRNA
scRNA
snoRNA
snRNA
SRP
tRNA
E
Adjusted p−value (−log10)
logFC (Cancer serum vs control serum)
RNA Type
lncRNA
other
rRNA
scRNA
snoRNA
snRNA
SRP
tRNA
