# Supplementary material for: Comprehensive transcriptomic analyses of tissue, serum, and serum exosomes from hepatocellular carcinoma patients
Source: BMC Cancer. 2019 Oct 28;19:1007. doi: 10.1186/s12885-019-6249-1 (PMC6816220; doi:10.1186/s12885-019-6249-1)
Supplement: Supplementary file 13 — Additional file 13: Figure S10. GO plot of miRNA target data. [file 12885_2019_6249_MOESM13_ESM.pdf]

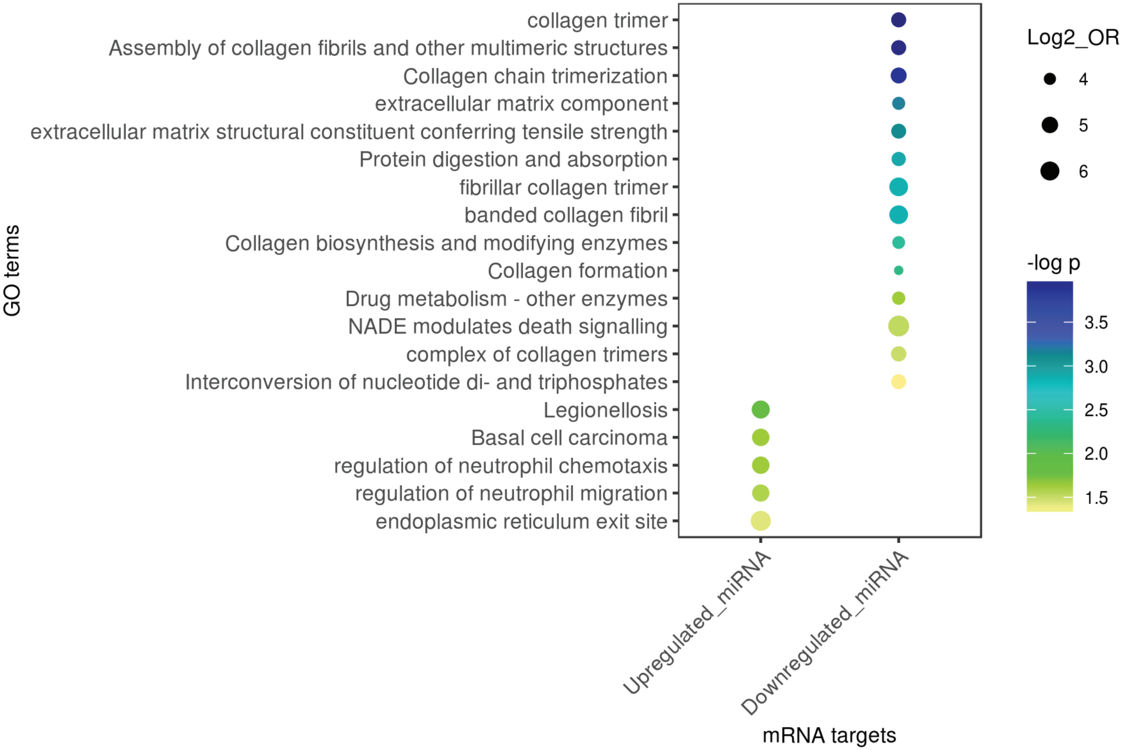
collagen trimer
Assembly of collagen fibrils and other multimeric structures
Collagen chain trimerization
extracellular matrix component
extracellular matrix structural constituent conferring tensile strength
Protein digestion and absorption
fibrillar collagen trimer
banded collagen fibril
Collagen biosynthesis and modifying enzymes
Collagen formation
Drug metabolism - other enzymes
NADE modulates death signalling
complex of collagen trimers
Interconversion of nucleotide di- and triphosphates
Legionellosis
Basal cell carcinoma
regulation of neutrophil chemotaxis
regulation of neutrophil migration
endoplasmic reticulum exit site
GO terms
Upregulated_miRNA
Downregulated_miRNA
mRNA targets
Log2_OR
4
5
6
-log p
3.5
3.0
2.5
2.0
1.5
